# The Cameroon Mobile Phone SMS (CAMPS) Trial: A Randomized Trial of Text Messaging versus Usual Care for Adherence to Antiretroviral Therapy

**DOI:** 10.1371/journal.pone.0046909

**Published:** 2012-12-06

**Authors:** Lawrence Mbuagbaw, Lehana Thabane, Pierre Ongolo-Zogo, Richard T. Lester, Edward J. Mills, Marek Smieja, Lisa Dolovich, Charles Kouanfack

**Affiliations:** 1 Centre for the Development of Best Practices in Health (CDBPH), Yaoundé Central Hospital, Yaoundé, Centre, Cameroon; 2 Department of Clinical Epidemiology and Biostatistics, McMaster University, Hamilton, Ontario, Canada; 3 Biostatistics Unit, Father Sean O'Sullivan Research Centre, St Joseph's Healthcare, Hamilton, Ontario, Canada; 4 Division of Infectious Diseases, Department of Medicine, University of British Columbia, Vancouver, British Columbia, Canada; 5 British Columbia Centre for Disease Control, Vancouver, British Columbia, Canada; 6 Faculty of Health Sciences, University of Ottawa, Ottawa, Ontario, Canada; 7 St. Joseph's Healthcare Hamilton, Ontario, Canada; 8 Department of Family Medicine, McMaster University, McMaster Innovation Park, Hamilton, Ontario, Canada; 9 Accredited Treatment Centre, Yaoundé Central Hospital, Yaoundé, Centre, Cameroon; Johns Hopkins University Bloomberg School of Public Health, United States of America

## Abstract

**Background:**

Mobile phone technology is a novel way of delivering health care and improving health outcomes. This trial investigates the use of motivational mobile phone text messages (SMS) to improve adherence to antiretroviral therapy (ART) over six months.

**Methodology/Principal Findings:**

CAMPS was a single-site randomized two-arm parallel design trial in Yaoundé, Cameroon. We enrolled and randomized HIV-positive adults on ART, aged 21 years and above to receive a weekly standardized motivational text message versus usual care alone. The primary outcome was adherence measured using a visual analogue scale (VAS), number of doses missed (in the week preceding the interview) and pharmacy refill data. Outcomes were measured at 3 and 6 months. Service providers and outcome assessors were blinded to allocation. Analysis was by intention-to-treat. Between November and December 2010, 200 participants were randomized, with 101 in the intervention group and 99 in the control group. At 6 months, overall retention was 81.5%. We found no significant effect on adherence by VAS>95% (risk ratio [RR] 1.06, 95% confidence interval [CI] 0.89, 1.29; p = 0.542; reported missed doses (RR 1.01, 95% CI 0.87, 1.16; p>0.999) or number of pharmacy refills (mean difference [MD] 0.1, 95% CI: 0.23, 0.43; p = 0.617. One participant in the intervention arm reported a possible disclosure of status.

**Conclusions/Significance:**

Standardized motivational mobile phone text messages did not significantly improve adherence to ART in this study. Other types of messaging or longer term studies are recommended.

**Registration:**

1. Pan-African Clinical Trials Registry; PACTR201011000261458

2. Clinicaltrials.gov; NCT01247181

## Introduction

There is increasing recognition for the potential of new technologies to improve health care, and the World Health Organization (WHO) prioritizes the use of new technologies to assist health delivery in resource-limited settings [1]. One technology that is widely used in resource-limited settings is the mobile telephone, as they are more reliable and less cumbersome than landlines [2].

Even though private ownership and use of mobile phones is not as widespread as in other more developed countries [3], Africa has shown great uptake of mobile phone technology [4]. For example, between 2000 and 2005 mobile phone subscriptions in Cameroon increased by 270% per annum [5]. In 2008, 37% of the adult population owned a mobile phone [6]. Given the aforementioned trend in mobile phone subscriptions it is reasonable to infer that a large majority of the adult population now own and use mobile phones. However, ownership is higher in urban areas [5].

The potential for text messages to improve health outcomes in resource limited settings is still being explored. In South Africa, SMS text messages have been used to improve HIV health care service delivery by improving communication between patients and health personnel, and also as an appointment reminder [7]. Two clinical trials in Kenya have evaluated the benefits of using mobile phone text message reminders to improve adherence to antiretroviral therapy (ART). The WelTel trial reported improvements in adherence and viral load [8], a second reported an improvement in adherence and a reduction in treatment interruptions [9]. A recent Cochrane systematic review summarized the evidence described in these two Kenyan trials [10]. The WHO recommends more research on adherence to long-term therapies because poor adherence leads to poor health and increased health costs [11]. However, the evidence on mobile phone text messaging to improve adherence to ART in developing countries is limited to one country (Kenya).

Given the importance of understanding the effectiveness of interventions to improve retention and adherence among people living with HIV in Africa, we conducted a randomized clinical trial to evaluate the utility of weekly motivational SMS texts on improving adherence and other important outcomes among a representative sample of HIV-positive adults in Cameroon.

### Objectives

The primary objective of our trial was to test the effectiveness of sending weekly motivational text messages via mobile phone versus no text messaging to improve adherence, measured using a VAS, the number of missed doses and pharmacy refills among HIV positive patients over a 6-month period at the Accredited Treatment Centre (ACT) of the Yaoundé Central Hospital (YCH). This is a busy urban treatment centre in Yaoundé, the capital city of Cameroon.

Our secondary objectives were to evaluate the effects on weight, body mass index (BMI), opportunistic infections (OI), CD4-positive-T-lymphocyte count, viral load, quality of life (QOL) measured using the SF-12 QOL assessment form [12], all-cause mortality, retention in care, adverse events and patient satisfaction. Subgroups of interest included age group, gender, level of education and treatment regimen.

## Methods

We report here a brief overview of the methods. Details can be obtained from the published protocol [13]. Using a parallel group design, eligible and consenting patients were randomized to intervention and control arms with a 1∶1 allocation ratio. Our findings are reported using the (CONsolidated Standards of Reporting Trials) CONSORT guidelines [14].The protocol for this trial and supporting CONSORT checklist are available as supporting information; see Checklist S1 and Protocol S1.

### Participants

Participants were recruited from the Yaoundé Central Hospital (YCH) Accredited Treatment Centre (ATC). The adult prevalence of HIV in Cameroon was 5.3% in 2009 [15]. The YCH is a referral hospital with a capacity of 381 beds, and staffed by 95 doctors and 270 nurses [16]. The ATC registers approximately 40 new cases per week and caters to approximately 6500 regular clients. It is the largest HIV/AIDS management clinic in Cameroon and enabled rapid recruitment.

We included subjects who were aged above 21 years; owned a mobile phone; who could read text messages; and who had been on ART for at least one month. Only those who provided informed consent, orally and in writing were allowed to participate.

We excluded individuals who had been on ART for less than one month at the time of enrollment, and were aged less than 21 years. Participants who had used ART for at least one month were chosen so that we could obtain a baseline adherence rate which we used to evaluate the success of randomization along with the other baseline covariates.

Participants were enrolled from the waiting rooms of the YCH ATC from the 22 November to the 22 December, 2010. The purpose of the trial was explained to consenting participants and baseline data were collected. Immediately after enrolment, trial codes and phone numbers were sequentially linked to predetermined allocation codes.

### Ethics

Ethical clearance was obtained from the Cameroon National Ethics Committee (authorization number 172/CNE/SE/2010). All participants included in the study provided both verbal and written consent.

### Interventions

We sent a short text message to each participant in the intervention (SMS) group, once a week, in either French or English, based on the participant's language preference. Messages were developed based on data collected from focus group discussions [17] and the health belief model of behavior change [18]. The content of the message was motivational, with a reminder component. The message also contained a phone number that they could call back if they needed help. The content was varied and contemporary (e.g. messages would contain season's greetings) so as to retain participants' attention throughout the study period and to explore the various aspects of behavior change. An example of a message would be, “You are important to your family. Please remember to take your medication. You can call us at this number: +237 xxxx xxxx.” The messages made no mention of HIV. We used a series of 11 messages that were changed every week. The program secretary used a list of phone numbers disclosed after randomization. One message was sent every week on Wednesdays at 9:00 am and the “delivery report” function of the mobile phone was used to determine if the message was actually received and opened. Text messaging was an add-on to usual care that includes regular ART counseling and home visits determined on a case-by-case basis.

In the control (no SMS) group, participants received only usual care. They did not receive any text messages, but they were interviewed at baseline, 3 months and 6 months. Data on satisfaction was collected only for the intervention arm, as it would have been inappropriate to ask people who did not receive text messages if they were satisfied with the intervention.

### Outcomes

Our primary outcome was adherence, measured using three methods: a Visual Analogue Scale (VAS); Self Report (SR); and Pharmacy Refill Data (PRD). Our Secondary outcomes were clinical: weight, body mass index (BMI), opportunistic infections (OI); QOL: Measured using the SF-12 QOL assessment form [12]; all cause mortality and retention in the trial.

### Sample size

Our study was designed to detect a 20% increase in adherence in the intervention arm. Sample size was calculated using WINPEPI (PEPI- for-windows) version 9.5 software [19]. Details of the assumptions used to arrive at a sample of 198, taking into account an attrition rate of 20% are reported in the protocol [13]. In brief, the study had 80% power to detect a statistically significant relative risk (alpha set at α = 0.05) using a two-tailed chi-squared test and assuming 60% and 80% adherence rates in the control and intervention groups respectively. Based on other studies using SMS to improve adherence [20] and reported adherence rates in Cameroon [21], it was estimated that at least a 20% increase in adherence was necessary to achieve adherence rates above 95%.

### Randomization, allocation concealment and implementation

Using a parallel group design, eligible and consenting patients were randomized to intervention and control arms with a 1∶1 allocation ratio. A computer generated randomization list was established using random block sizes of 2, 4 and 6, by the Father Sean O'Sullivan Research Centre Biostatistics Unit at St Joseph's Healthcare/McMaster University (http://www.thecem.net/sjhsrn.php) in Canada. The allocation codes were then sequentially affixed to the phone numbers of consecutively recruited participants by trained research staff at the YCH ATC. This sequence was sent to the research centre by email, and concealed in a password-protected computer until interventions were assigned.

### Blinding

Trained interviewers – blinded to group allocation – collected data using a pre-tested data collection form containing socio-demographic data, clinical information and adherence rates at baseline, 3 and 6 months. From the point of enrollment, patients were identified only by their phone numbers and their sequential trial numbers. The interviewers transmitted the phone numbers of the enrollees to the research staff. The research staff responsible for allocation had access to the allocation codes and the phone numbers of participants. The program secretary responsible for sending the text messages received the allocations (SMS or No SMS) and corresponding phone numbers weekly. The data analyst was also blinded to group allocation. Only the participants were aware of their allocation.

### Statistical methods

We adopted the intention-to-treat principle to analyze all outcomes, meaning that data from participants was analyzed according to the group to which they were randomized irrespective of whether they actually received the intervention. We also used multiple imputation techniques to handle missing data [22]. Variables for which there was too much missing data to perform imputation were excluded from the analysis but are reported (CD4-T-lymphocyte cell count and viral load). All outcome variables had some degree of missing data ranging from 0 to 35%. Multiple imputation was used to create a new data set which was the average of five data sets of imputed values. This final data set was used for all analyses. We used the t-test to compare groups on continuous outcomes and the chi-squared test for binary outcomes. All statistical tests were performed using two-sided tests at the 0.05 level of significance. The Bonferroni method was used to adjust the level of significance for testing of secondary outcomes. For group comparisons, the results are expressed as mean difference (MD) and risk ratio (RR) for binary outcomes, corresponding two-sided 95% confidence intervals (95% CIs) and associated p-values. Adjusted analyses using baseline covariates (age, gender, educational level, duration on ART, HIV staging, BMI and the presence of an OI) were performed using standard binary logistic regression techniques to investigate the residual impact of these characteristics on the primary outcome. We included the interaction term for the intervention variable and the following covariates: age group, gender, level of education and regimen. These covariates are reported to affect adherence rates to ART [11]. Goodness-of-fit was assessed by examining the residuals for model assumptions and the Hosmer and Lemeshow test of goodness-of-fit. The p-values for the interaction terms are reported. All analyses were performed using SPSS (Statistical Package for the Social Sciences) version 16.0 for Windows and WINPEPI [19].

## Results

### Recruitment, baseline data and participant flow

**Figure 1:**
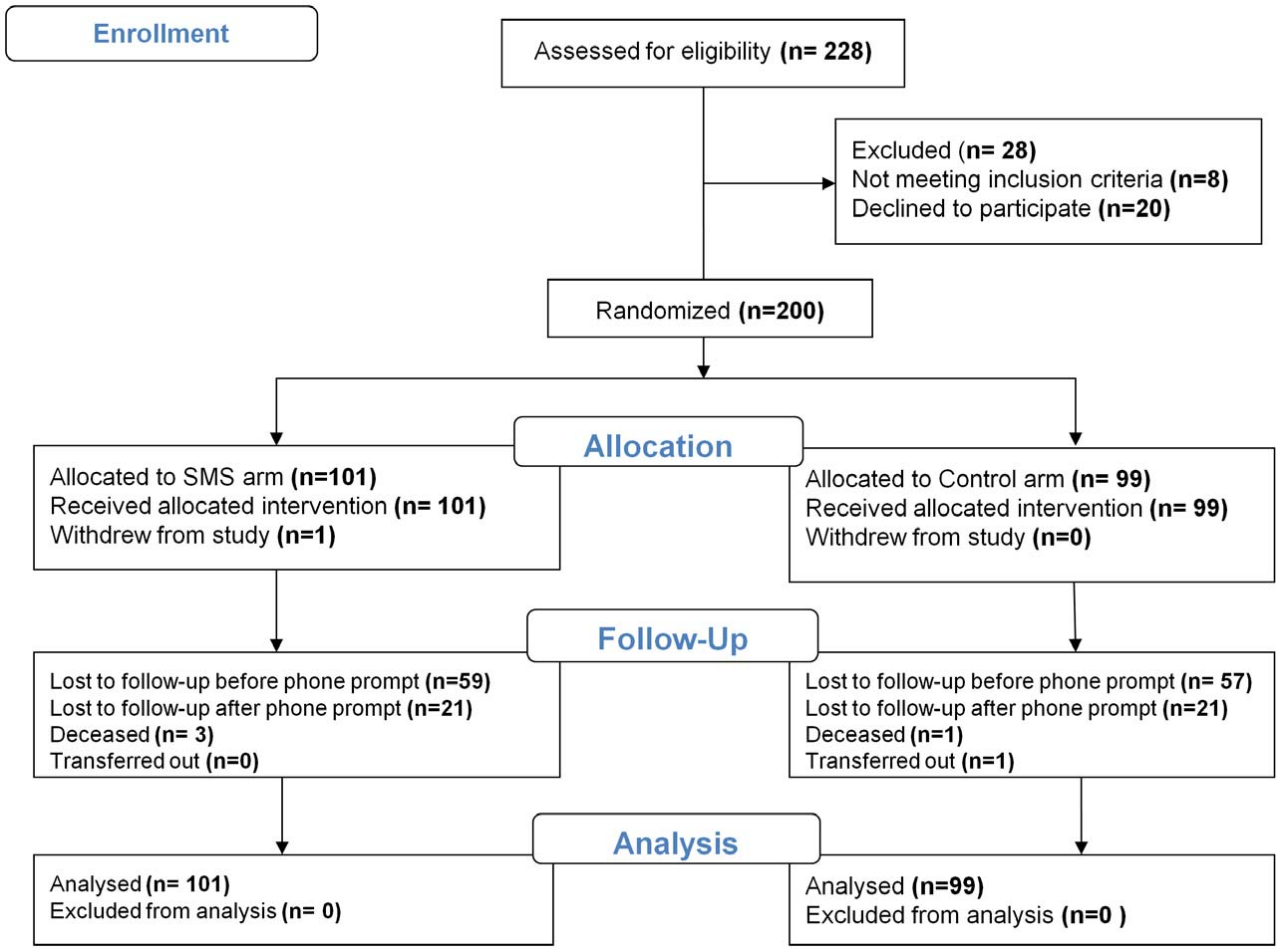
CONSORT flow diagram for CAMPS trial.

Between November and December 2010, 228 patients were approached for enrollment. Twenty declined to participate and 8 did not meet the eligibility criteria. Two hundred participants were randomized to either the SMS intervention arm (n = 101) or the control arm (n = 99). One participant in the intervention arm withdrew due to loss of privacy. Initial retention in the trial for both arms at 6 months was 42% (participants who came for scheduled clinic visits), but increased to 82% (after a phone call inviting them to come for a final interview). Participants were followed up from December 2010 to May 2011, when the intervention was stopped. During this period, we received 99 phone calls and 55 text messages (154 responses) from 48 participants in the intervention arm. The content of these responses is the subject of another manuscript. Figure 1 displays the flow of participants in the study. Data for all 200 participants were analyzed. After randomization, both groups were similar in baseline characteristics (table 1).

**Table 1:** Demographics and baseline.

Variable	SMS group (n = 101)	Control group (n = 99)
**Age (years): mean (SD)**	41.3 (10.1)&	39.0 (10.0)&
**Gender:** n (%)		
Female	69 (68.3)	78 (78.8)
**Level of education:** n (%)		
None	1 (1.0)	3 (3.0)
Primary	42 (41.6)	32 (32.3)
Secondary	46 (45.5)	51 (51.5)
Tertiary	12 (11.9)	13 (13.1)
**Family aware of HIV status:** n (%)	88 (87.1)	92 (92.9)
**Presence of an opportunistic infection:** n(%)	36 (35.6)	26 (26.3)
**BMI : mean (SD)**	25.3 (4.1)ε	25.2 (4.0)β
CDC* classification - AIDS defining illness§:n(%)	76 (75.2)α	70(70.7)δ
**Regimen:** n (%)		
First line	91 (90.1)	88 (88.9)
Second line	7 (6.9)	11 (11.1)
**Duration on ARV (months): median (Q1, Q3)**	31.0 (15.0, 50.5)	22.0 (7.0,46.0)μ
**CD4 (cells per mm^3^): median (Q1, Q3)**	347.0 (211.0, 527.5)	327.0 (194.0,475.0)
**Adherence (Visual Analogue Scale): mean (SD)**	88.8 (13.42)ε	92.4 (11.84)δ

& = 1 missing;

β = 8 missing;

ε = 6 missing;

α = 3 missing;

δ = 4 missing;

μ = 2 missing;

SD: standard deviation; CDC: Centres for Disease Control, § CDC classifications: A3, B3, C1, C1, C3 [1]; CD4: CD4-positive-T-lymphocyte; Q1: first quartile; Q3: third quartile.

### Outcomes and estimation

At 6 months, we found no effect on the number of participants achieving >95% adherence by VAS (RR 1.06, 95% CI 0.89, 1.29; p = 0.542) or reporting missed doses (RR 1.01, 95% CI 0.87, 1.16; p>0.999). The mean number of pharmacy refills was also not different between groups (mean difference [MD] 0.1 95% CI −0.23, 0.43; p = 0.617). However, on sensitivity analysis, more participants in the SMS group achieved adherence of >90% at 6 months (RR 1.14 95% CI 1.01, 1.29; p = 0.027). The details for the other secondary outcomes at 6 months are reported in table 2.

**Table 2:** Outcomes at 6 months.

Outcome	Type	SMS group (n = 101)	Control group (n = 99)	Effect Estimateμ
**Primary**	**Binary**	**n (%)**	**n (%)**	**RR (95%CI);p**
	VAS>95%	72 (71.3)	66 (66.7)	1.06 (0.89,1.29; 0.542
	Self report (no missed doses)	80 (79.2)	78 (79.0)	1.01 (0.87,1.16;) >0.999
	**Continuous**	**Mean (SD)**	**Mean (SD)**	**MD (95% CI);p**
	Pharmacy Refill Data	3.8 (1.48)	3.7 (1.34)	0.1 (−0.23,0.43); 0.617
**Secondary** * ¥	**Binary**	**n (%)**	**n (%)**	**RR (95%CI);p**
	VAS>90%	91 (90.1)	78 (78.8)	1.14 (1.01,1.29);0.027
	Presence of a new OI	20 (19.8)	17 (17.2)	1.15 (0.64,2.07); 0.632
	Mortality	3 (2.9)	1(1.0)	2.94 (0.31–27.79); 0.322
	Retention	80 (79.2)	83 (83.8)	0.95 (0.83,1.08); 0.399
	**Continuous**	**Mean (SD)**	**Mean (SD)**	**MD (95% CI);p**
	Weight (kg)	71.8 (11.97)	70.2 (11.87)	1.60(−1.72,4.92); 0.344
	BMI	26.54 (4.254)	25.73(3.823)	0.81(−0.32,1.94); 0.159
	Quality of life (SF-12 scale score)	3.79 (0.585)	3.75 (0.583)	0.04(−0.12,0.20); 0.629

(SMS: short message service; RR: risk ratio; CI: confidence interval; SD: standard deviation; MD: mean difference; VAS: visual analogue scale; BMI: body mass index; OI: opportunistic infection; CD4: CD4-positive-T-lymphocyte; SF: short form).

* Bonferroni adjustment for secondary outcomes: 0.05/8 = 0.006.

¥ Insufficient data for CD4 count (n = 34 for intervention and 26 for control; MD-24.4; 95% CI: −101.3, 52.6; p = 0.599) and viral load (n = 0).

μ P-values obtained using the chi-squared test and the t-test for binary and continuous outcomes respectively.

At 3 months, fewer participants in the SMS group had an adherence rate of >95% (RR 0.77, 95% CI 0.63, 0.94; p = 0.029) or >90% (RR 0.61 95% CI 0.32, 1.14; p = 0.094); equal numbers reported missed doses (RR = 0.97, 95% CI 0.85, 1.10; p = 0.622), and the mean number of pharmacy refills was not significantly different (MD 0.10, 95% CI −0.03, 0.23; p = 0.139). The other secondary outcomes at 3 months are reported in table 3.

**Table 3:** Outcomes at 3 months.

Outcome	Type	SMS group (n = 101)	Control group (n = 99)	Effect Estimateμ
**Primary**	**Binary**	**n (%)**	**n (%)**	**RR (95%CI);p**
	VAS>95%	52(51.5)	66(66.7)	0.77(0.63,0.94);0.029
	Self report (no missed doses)	82 (81.2)	83(83.8)	0.97(0.85,1.10);0.622
	**Continuous**	**Mean (SD)**	**Mean (SD)**	**MD (95% CI);p**
	Pharmacy Refill Data	2.3(0.50)	2.2 (0.45)	0.10 (−0.03,0.23);0.139
**Secondary** * ¥	**Binary**	**n (%)**	**n (%)**	**RR (95%CI);p**
	VAS>90%	60(59.4)	70(70.7)	0.61(0.32,1.14);0.094
	Presence of a new OI	39(38.6)	32(32.3)	1.19 (0.82,1.74);0.353
	Mortality	0	0	Not estimable
	Retention	85(84.2)	84(84.8)	0.99(0.88,1.12); 0.893
	**Continuous**	**Mean (SD)**	**Mean (SD)**	**MD (95% CI);p**
	Weight (kg)	70.9(12.18)	71.2(12.96)	−0.30 (−3.81,3.21);0.866
	BMI	26.24(4.087)	26.07(4.175)	0.17 (−0.98,1.32);0.771
	CD4 (cells per mm^3^):	406 (230)	375 (225)	31 (−32.5,94.5); 0.337
	Quality of life (SF-12 scale score)	3.67 (0.623)	3.69 (0.615)	−0.20(−0.19,0.15);0.820

(SMS: short message service; RR: risk ratio; CI: confidence interval; SD: standard deviation; MD: mean difference; VAS: visual analogue scale; BMI: body mass index; OI: opportunistic infection; CD4: CD4-positive-T-lymphocyte; SF: short form).

* Bonferroni adjustment for secondary outcomes: 0.05/8 = 0.006.

¥ Insufficient data for viral load (n = 0).

μ P-values obtained using the chi-squared test and the t-test for binary and continuous outcomes respectively.

### Ancillary analyses

#### Regression

We performed regression analyses to determine the impact of baseline covariates on the primary outcomes. Higher levels of education (OR = 5.32, 95% CI 2.51, 11.30; p<0.001) and being on a second line regimen (OR = 11.06, 95% CI 3.75, 32.65, p<0.001) were statistically significant predictors of adherence >95%. The Hosmer and Lemeshow goodness-of-fit test was as follows: chi-squared 56.7, degrees of freedom = 8, p<0.001.

We also added an interaction term between covariates and the intervention variable. The interaction terms were not statistically significant for age group (p = 0.633) and gender (p = 0.268), but statistically significant for level of education (p<0.001) and regimen (p<0.001).

#### Adverse events

One female in the intervention arm requested to withdraw from the study because she felt it had compromised her undisclosed status. No other undesirable effects were reported.

#### Satisfaction

Satisfaction with the text message was measured using four questions (Table 4): rating of the SMS; if it helped improve adherence; if they wanted it to continue and if they would recommend it to a friend. Moderate levels of satisfaction (65% reported that the messages were good, very good or excellent) were reported by the participants who received the text messages.

**Table 4:** Satisfaction with the text message among the participants who received text messages (n = 101).

Question	Count (%)*
**How would you rate the text message?**	
Excellent	12 (11.8)
Very good	30 (29.7)
Good	21(20.8)
Average	17 (16.8)
Bad	5 (4.9)
Very bad	16 (15.8)
**Did it help you remember to take your medication?**	
Yes	92 (91.1)
No	9 (8.9
**Do you want to continue receiving text messages?**	
Yes	66 (65.3)
No	35 (34.7)
**Would you recommend it to a friend?**	
Yes	82 (81.2)
No	19 (18.8)

* Percentages may not add up to 100 due to rounding off.

## Discussion

Our study did not find a significant effect of motivational SMS texts on improving adherence to ART over a 3 to 6 month period. This trial was unique in that it was the first to report the effect of an SMS intervention on ART adherence among treatment experienced patients in Africa. Two recent trials in Kenya demonstrated improved ART adherence among patients initiating ART, but used different intervention protocols [8], [9].

Our study had some important limitations. Firstly, our primary measures of adherence (by interviews) might have resulted in overestimates of the true adherence rate [23], [24] and the adherence reported for the last week may not adequately reflect adherence behaviors over longer periods because patients may become more adherent in the few days preceding their appointment [25]. However, pharmacy refill data showed similar findings to self-reports. The trial design did not interfere with patient care by providing medication or lab tests so there are large amounts of missing data for CD4-positive-T-lymphocyte count and viral load. Drug stock-outs were also frequent in the last two months of the trial. This may explain why some participants missed their scheduled appointments. Importantly, participants in the control arm were not prohibited from using other reminder methods, so additional benefits may have been difficult to detect. Previous studies conducted in the same parent population suggest that up to 25% have systematic reminder methods [17]. Finally, our sample size was powered to detect a 20% difference in adherence between both arms. The difference we found was much less.

Our findings should be interpreted in light of the published trials in Kenya [8], [9], which show some improved adherence rates after twelve months, also reported in a Cochrane review synthesizing data from both trials [10]. The interventions evaluated were somewhat different. While our trial used motivational messages, with the intention to produce a change in adherence behavior, and no compulsory feedback, the Weltel trial used a simple SMS inquiry on the participants' health and was therefore interactive [8]. The second trial used short and long one-way messages: the longer message with encouraging content, but no option for feedback [9]. Even though we used optional feedback, we did not detect any improvements in adherence. Only 48 participants in the intervention arm used the feedback option. We also used weekly messages like the Kenyan trials, but did not observe any significant benefits. Both Kenyan trials ran for up to one year, while our trial ended at 6 months. The duration of our trial might not have been sufficient to observe a significant effect.

Another important difference is the fact that both Kenyan trials enrolled participants who had recently initiated ART [8], [9]. The median duration on ART at baseline in this study was 31 and 22 months for the intervention and control groups respectively. This may also explain the negative results, since duration on ART has been shown to have negative effects on adherence to ART in Cameroon [26]. We speculate that the SMS may be more effective in treatment-naïve populations. While the risk of disclosure of status has been mentioned in some studies [27], this is the first study documenting a case of withdrawal for privacy reasons. In Cameroon, there is still a lot of stigma associated with HIV, and it is a known cause of poor adherence [28]. Although we did not include the term “HIV” in the content of the text messages, we did include “medications” and gave a clinic number which could arouse suspicion by non-participants reading the message. Interestingly, we had a very high proportion of clients in this study who reported having disclosed their status to their families. This may have reflected a selection bias for enrolment, and larger benefits may have been observed in individuals who do not realize the support of disclosing. Confidentiality and disclosure are important considerations for the scale-up of text message interventions [29].

High levels of satisfaction have been documented in other text message trials, particularly in those which offer two way communication [8], [30]. While the majority of participants in our trial were satisfied with the text messages, a considerable number did not want the intervention to continue. A study conducted prior to this trial reported that patients would like to receive messages with a wide variety of characteristics in terms of timing, content and source [17]. Some participants might not have wanted to continue if the messages weren't tailored to their needs. Yet, more than 80% would recommend it to their friends. Further research is needed on how best to tailor text messages. It is unclear whether the content of the message played a role in the outcomes, as other trials with no motivational component have reported improvements in adherence [8], [9].

The ancillary analyses reported above need to be considered as secondary and therefore interpreted with caution in the light of our main findings.

In conclusion, motivational text messages did not significantly improve adherence to ART among treatment experienced patients in Cameroon after 6 months. Although interactive SMS associated with access to health advice has demonstrated to be effective in at least one large clinical trial [8], and is reflected in current guidelines [31] more work needs to be done to determine how motivational content can be delivered by SMS alone. Text messages may come with a small risk of disclosure of status. Further trials are critical to determine what interventions should be taken to scale.

## Supporting Information

Protocol S1 **Research Protocol.**
(PDF)

Checklist S1 **CONSORT checklist.**
(DOC)
