# Supplementary material for: The Cameroon Mobile Phone SMS (CAMPS) Trial: A Randomized Trial of Text Messaging versus Usual Care for Adherence to Antiretroviral Therapy
Source: PLoS One. 2012 Dec 6;7(12):e46909. doi: 10.1371/journal.pone.0046909 (PMC3516507; doi:10.1371/journal.pone.0046909)
Supplement: Protocol S1 — Research Protocol. (PDF) [file pone.0046909.s001.pdf]

Improving Adherence to HAART using mobile text messages in a low resource setting: A randomized controlled trial

(Research Protocol) Version: 1.2

Principal Investigator:

Lawrence Mbuagbaw

Trial site Coordinator:

Kouanfack Charles

Sponsor:

Center for Development of Best Practices in Health

Yaoundé Central Hospital

Supervisors:

Prof Thabane Lehana

Prof Jimmy Volmink

Dr Pierre Ongolo-Zogo

Steering Group:

Dr Yondo David, Renee Cecile Bonono, Dr Marie-Jose Essi, Dr Mba Robert, Dr Serge Ndongo

Table of Contents

1. List of Abbreviations
2. Background
3. Objectives and purpose
4. Design
5. Selection and exclusion of subjects
6. Assessment of efficacy
7. Assessment of safety
8. Discontinuation of study
9. Statistics
10. Procedure for accounting for missing, unused, and spurious data
11. Ethics
12. Data handling and record keeping
13. Project timetable
14. Responsibilities of researchers
15. References
16. Appendix

1. List of Abbreviations

ART	Antiretroviral Treatment
BMI	Body Mass Index
CD4	Cluster Designation 4
CDC	Centre for Disease control
HAART	Highly Active Antiretroviral Treatment
HIV	Human Immuno-deficiency Virus
MEMS	Micro-Electronic Memory system
OI	Opportunistic Infection
PRD	Pharmacy Refill Data
SMS	Short Message Service
SPSS	Statistical Package for Social Sciences
SR	Self Report
VAS	Visual Analogue Scale
WHO	World Health Organization

2. Background

Mobile text messages using the Short Message Service (SMS) are a cheap and non-invasive means of communication that can be used to convey health related messages to owners of mobile phones. There is contradictory evidence concerning the role of mobile phones in ameliorating health outcomes, especially in less developed countries where private ownership and use of mobile phones is not as widespread as in other more developed countries.[(1)] However, mobile phone calls and reminders have been demonstrated to improve adherence to care of patients with tuberculosis. [(2)] Africa has the greatest uptake of mobile phone technology. [(3)] Cameroon benefits from the availability of multiple mobile operators, and the SMS is already used for business transactions, personal communication, advertising and betting. Cameroon already has mobile phone ownership coverage of about 60%. There is a potential for new benefits to be discovered in the use of mobile phone technology in health interventions for resource- limited countries. [(4)] Radiofrequency exposure due to prolonged mobile phone use is concentrated in the tissues closest to the phone. One study found an increased risk in acoustic neuroma's after ten years of phone use. [(5)] We found no data on risk associated with using mobile phone use for text messages.

The advent of antiretroviral treatment (ART) has markedly reduced morbidity and mortality associated with the Human Immunodeficiency Virus (HIV). [(6)] Much effort has been put into the scaling up of access to ART. [(7)] The efficacy of ART depends largely on compliance to treatment regimens. Poor adherence is associated with poor virological and immunological response. It is also responsible for the development of resistant strains. [(8)]

Other studies on using mobile phone technology to improve adherence to ART discouraged the use of phone calls as they are time and labor intensive.[(9)] Cost will also be an issue in a low resource setting. Another study reported high satisfaction with two way text messaging. [(10)] Both of these studies were performed in developed countries. A third study, investigating the use of text messaging to improve adherence to primary care found that it was more cost effective than phone calls.[(11)] In South Africa, the SMS has been demonstrated to improve HIV health care service delivery by ameliorating communication between health workers and patients and also as an appointment reminder. [(12)]

3. Trial Objectives and Purpose

The purpose of this trial is to investigate the efficacy of mobile phone text messages in improving adherence to Highly Active Antiretroviral Treatment (HAART) in a low resource setting. Our findings will improve the body of evidence on devices aimed at improving adherence not only to HAART, but also to all other long term treatments.

Our objectives are to:

- Compare the rates of adherence in patients receiving SMS reminders and patients receiving standard care.
- Compare changes in Cluster Designation 4 (CD4) count and viral load in patients receiving SMS reminders and patients receiving standard care.
- Compare the changes in HIV staging in patients receiving SMS reminders and patients receiving standard care.
- Compare the mortality in patients receiving SMS reminders and patients receiving standard care.
- Compare satisfaction with care
- Compare quality of life between patients receiving SMS reminders and patients receiving standard care.

4. Trial Design

We will perform a randomized controlled single blind trial, with two arms. The patients will be randomly allocated to each arm. The patients in one arm will receive routine care and a weekly motivational reminder text message; and the patients in the other arm will receive only routine care.

Endpoints:

Our primary endpoint will be adherence rates, measured by self report, the visual analogue scale and pharmacy refill data.

Our Secondary endpoints will be;

- Social: Satisfaction with care, retention in care, quality of life
- Clinical: Weight, Body Mass Index (BMI), Opportunistic Infections(OI), WHO (World Health Organization) Classification [13], mortality
- Biological: CD4 count, viral load
- Composite: Center for Disease Control (CDC) Classification [14]

Randomization:

This is a parallel group design evaluating the effects of adding daily SMS text messages using mobile phones to usual care (intervention) versus usual care alone (control) among HIV positive patients on HAART. Eligible and consenting patients will be randomized to intervention and control arms using 1:1 allocation ratio by opaque sealed envelope method. A computer generated randomization list will be generated using random block sizes of 2, 4 and 6, by the Father Sean O'Sullivan Research Centre Biostatistics Unit at St Joseph's

Healthcare/McMaster University. The allocation codes will then be put in sequentially numbered opaque sealed envelopes and administered by the trained Research Staff at YCH ATC centre. Trained interviewers – blinded to group allocation – will collect data using a pretested data collection form containing socio-demographic data, clinical information and adherence rates at baseline, 3 and 6 months. The data analyst will also be blinded to group allocation.

Intervention:

We will send a short text message to the participants in the intervention group as follows:" This is a reminder to take your medication." The person in charge of sending the text messages will use the delivery report function to ensure that the message has been delivered.

Duration:

The trial will run for six months, with interim analysis at 3 months.

Discontinuation:

Individual participants are free to discontinue their participation in the trial at any time. We will endeavor to elucidate their reasons for withdrawal.

Accountability:

A register of all text messages sent will be kept.

Maintenance of trial treatment randomization codes and procedures for breaking codes:

The randomization codes shall be maintained until the end of the study and broken only during data analysis.

Sources of data:

We will use data from hospital files and personal medical records to fill the case record forms in addition to structured interviews. See appendix for data collection form.

5. <u>Selection and Exclusion of Subjects:</u>

Subject inclusion criteria:

- Age 21 and above
- Owns a mobile phone and can read text messages
- On HAART for at least a month

Subject exclusion criteria:

- On HAART for less than one month
- Doesn't fulfill eligibility criteria
- Refuses to take part

6. Assessment of Efficacy

Efficacy will be measured using the Visual Analogue Scale (VAS), Pharmacy Refill Data (PRD), and Self Report (SR). The VAS is highly correlated with more objective methods like using Microelectronic Monitoring System caps (MEMS). [15] The VAS scale is a simple tool that measures attributes that range along a continuum. It may be presented as a horizontal or vertical line, with maximum and minimum values of the attribute to be measured on either extremity. The subject locates his position along the line with respect to the minimum or maximum values.

Efficacy will be assessed at 3 months (without unblinding) and at six months.

7. Assessment of Safety

We will determine participant satisfaction with the intervention and note any unwanted effects of weekly text messages.

8. Discontinuation of the Study

The study may be discontinued if the data suggests that there are no more benefits to be achieved by continuing the study.

9. Statistics

The sample size calculation is based on the test of the null hypothesis that the rates of adherence to HAART in the two groups (intervention and control) are equal. The primary measure of effect is the rate of adherence to ART treatment as measured by using the VAS over 6 months. The criterion for significance (alpha) has been set at 0.05. The test is 2-tailed, which means that an effect in either direction will be interpreted. The sample size was calculated using the WINPEPI (PEPI- for-windows) version 9.5 software [16]. With the proposed sample size of 82 in each of the two groups (i.e. assuming a 1:1 allocation ratio), the study will have power of 80% to yield a statistically significant result using a chi-squared test (assuming an intention-to-treat principle for the analysis) of the relative risk at alpha = 0.05. This computation assumes an adherence rate of 80% (for the intervention group) versus 60% (for the control group) at 6 months. These estimates are reflective of estimates from similar studies investigating SMS effect on drug adherences [17] and were modified to account for the type of intervention and patients for this study. We adjusted the sample size for a potential attrition rate of 20% (due to drop-outs) based on attrition rates to care in this centre. Therefore, the

required sample size is 198 patients (99 per group). At the YCH ATC, on average, there are about 120 patients put on HAART per month. We estimate that about 90% will have mobile phones and would be eligible to participate in the study. Of these, it is expected that approximately 75% would be willing to participate in the study and will provide consent to participate in the trial. The expected period for recruitment will be one month to obtain 198 patients needed for the trial. It is feasible to recruit 198 patients in one month because we will also recruit from the large pool of old patients. Our study is designed to detect a 20% increase in adherence.

We will analyze the data by comparison of two groups using two sided chi-squared tests for binary outcomes and the T test for continuous outcomes. Gender and educational level are potential subgroups of interest. Treatment arms will be compared with respect to the following covariates: age, gender, education, duration on HAART, HIV staging, treatment regimen.

We will use logistic regression for the multivariate analysis of binary outcomes and linear regression for the continuous outcomes. Relative risks will be calculated with 95% confidence intervals. P values will be reported. Data will be analyzed using the Statistical Package for Social Sciences (SPSS) Version 17.0.

Our outcome measures are listed in table 1.

Table 1: Overview of outcome measures

**Outcome measures**	**Scale**	**Type**	**Measure**	**Analysis method**
**Primary**				
Adherence at 6 months				
VAS	Ordinal	Binary	VAS percentage >95%	Chi-squared test
Self report	Ordinal	Binary	% adherence in last month>95%	Chi-squared test
PRD	Ordinal	Binary	% of complete refills <	Chi-squared test
**Secondary**				
Weight	Ratio	Continuous	Change in weight	T-test
BMI	Ratio	Continuous	Change in BMI	T-test
OI	Nominal	Binary	Occurrence of new OI	Chi-squared test
WHO Classification	Ordinal	Categorical	Change in classification	Chi-squared test
CDC Classification	Ordinal	Categorical	Change in classification	Chi-squared test
Mortality	Nominal	Binary	All deaths	Chi-squared test
Retention	Nominal	Binary	Number retained in care	Chi-squared test
CD4 count	Ratio	Continuous	Change in CD4 count	T-test
Viral load	Ratio	Continuous	Change in viral load	T-test
Satisfaction with care	Ordinal	Categorical	Change in satisfaction scores	T-test
QoL	Ordinal	Categorical	Change in QoL scores	T-test

(VAS: visual analogue scale, PRD: pharmacy refill data, BMI, body mass index, OI: opportunistic infection, WHO: World Health Organization, CDC: Center for Disease Control, CD4: Cluster Designation 4, QoL: Quality of Life)

10. **Additional studies:**

- **Safety and patient satisfaction:** Data will be collected on issues that may arise from the use of text messages to improve adherence.
- **Health worker experiences:** Self-administered questionnaires will be used to assess health worker perceptions of the intervention in terms of long term use, additional workload and benefits to care.

11. **Procedure for accounting for missing, unused, and spurious data**

We will perform our analysis on an intention to treat basis. Missing data will be reported as such. Any deviations from the statistical plan described in the protocol will be described and justified in the final report. All randomized subjects will be included in the analysis.

12. **Ethics**

The study will be conducted in accordance with the Helsinki declaration and established guideline for research on human subjects. All participants must provide informed consent prior to enrollment. See informed consent form. Post trial care with text messages will not be provided to participants and they will be informed of this. The National Ethics Committee will be immediately informed of any changes made to the protocol.

13. **Data Handling and Recordkeeping**

The interviewers will be trained on data collection. Case report forms will be checked by the principal investigator and saved in electronic format. The hard copies will be safeguarded in a secure location. The National Ethics Committee will have access to source data for trial-related monitoring purposes.

14. **Project Timetable/Flowchart**

Table 1: Overview of study timetable

Month	Activity
1	Enrollment, randomization, allocation
2	Supervision
3	Supervision
4	Follow up, interim analysis
5	Supervision

**6**	**Supervision**
**7**	**End of study, final analysis, write-up**

15. Responsibilities of researchers:

The principal investigator is responsible for the overall running of the study, obtention of ethical clearance, protocol development, logistics and methodological issues.

The study site coordinator is responsible for the day to day running of the trial, interviewer supervision, ensuring that enrollment is going on according to plan.

The supervisors oversee the activities of the principal investigator and the study site coordinator. They provide mentorship.

16. References

[1] Warren A Kaplan. Can the ubiquitous power of mobile phones be used to improve health outcomes in developing countries? Globalization and Health 2006, 2:9doi:10.1186/1744-8603-2-9

[2] Liu Q, Abba K, Alejandria MM, Balanag VM, Berba RP, Lansang MAD. Reminder systems and late patient tracers in the diagnosis and management of tuberculosis. Cochrane Database of Systematic Reviews 2008, Issue 4. Art. No.: CD006594. DOI: 10.1002/14651858.CD006594.pub2. Evidence Update published in June 2010.

[3] Lester R T, Gelmon L and Plummer FA: Cellphones: tightening the communication gap in resource-limited antiretroviral programmes? Aids 2006, 20:2242–2244.

[4] Lester R and Karanja S: Mobile phones: exceptional tools for HIV/AIDS, health, and crisis management. Lancet Infect Dis 2008, 8:738–739.

[5] Mobile phone use and the risk of acoustic neuroma. JSTOR Epidemiology 2004; 15(6): 653-659.

[6] Mocroft A, Vella S, Benfield TL, Chiesi A, Miller V, Gargalianos P et al. Changing patterns of mortality across Europe in patients infected with HIV-1. EuroSIDA Study Group. Lancet 1998 Nov;28;352(9142):1725-30.

[7] UNAIDS 2009. Report on global AIDS epidemic. http://data.unaids.org/pub/Report/2009/2009_epidemic_update_en.pdf. (accessed 15/12/2009)

[8] Gill CJ, Davidson HH, Jonathon LS et al. No Complacency about adherence to antiretroviral therapy in sub-saharan Africa. AIDS 2005; 19:1243-1249.

[9] Wiener L, Riekert K, Ryder C, Wood LV: Assessing medication adherence in adolescents with HIV when electronic monitoring is not feasible. AIDS Patient Care STDS 2004, 18:527-538.

[10] Dunbar PJ, Madigan D, Grohskopf LA, Revere D, Woodward J, Minstrell J, Frick PA, Simoni JM, Hooton TM: A two-way messaging system to enhance antiretroviral adherence. J Am Med Inform Assoc 2003, 10:11-15.

[11] Leong KC, Chen WS, Leong KW, Mastura I, Mimi O, Sheikh MA, Zailinawati AH, Ng C J, Phua KL and Teng CL. The use of text messaging to improve attendance in primary care: a randomized controlled trial. Family Practice 2006; 23: 699–705.

[12] Mukund Bahadur KC, Murrayb PJ: Cell Phone Short Messaging Service (SMS) for HIV/AIDS in South Africa: A literature review. Stud Health Technol Inform 2010, 160:530-534.

[13] World Health Organization. WHO Case Definitions of HIV for Surveillance and Revised Clinical Staging and Immunological Classification of HIV-Related Disease in Adults and Children. 2007. Accessed October 15, 2010. Available at URL: [www.who.int/hiv/pub/guidelines/HIVstaging150307.pdf].

[14] Centers for Disease Control and Prevention. 1993 revised classification system for HIV infection and expanded surveillance case definition for AIDS among adolescents and adults. MMWR Recomm Rep. 1992 Dec 18;41(RR-17):1-19. Accessed October 15, 2010. Available online at URL: [www.cdc.gov/mmwr/preview/mmwrhtml/00018871.htm]

[15] Arnsten JH, Demas PA, Farzadegan H, Grant RW,Gourevitch MN, Chang CJ, Buono D, Eckholdt H, Howard AA,Schoenbaum EE. Antiretroviral therapy adherence andviral suppression in HIV-infected drug users: comparisonof self-report and electronic monitoring. Clin Infect Dis 2001; 33:1417-23.

[16] Abramson, J.H: **WINPEPI (PEPI-for-Windows): computer programs for epidemiologists.** Epidemiologic Perspectives & Innovations 2004, **1:** 6.

[17] Strandbygaard U, Thomsen SF, Backer V: **A daily SMS reminder increases adherence to asthma treatment: a three-month follow-up study.** Respir Med 2010, **104:**166-171.
